# Effectiveness of the tailored EBP training program for Filipino physiotherapists: A randomised controlled trial

**DOI:** 10.1186/1472-6920-11-14

**Published:** 2011-04-13

**Authors:** Janine Margarita Dizon, Karen Grimmer-Somers, Saravana Kumar

**Affiliations:** 1 International Centre for Allied Health Evidence University of South Australia City East Campus, North Terrace Adelaide 5000, Australia; 2 University of Santo Tomas Manila 1015 Philippines

## Abstract

**Astract:**

**Background**

Evidence implementation continues to challenge health professionals most especially those from developing countries. Filipino physiotherapists represent a group of health professionals in a developing country who by tradition and historical practice, take direction from a doctor, on treatment options. Lack of autonomy in decision-making challenges their capacity to deliver evidence-based care. However, this scenario should not limit them from updating and up-skilling themselves on evidence- based practice (EBP). EBP training tailored to their needs and practice was developed to address this gap. This study will be conducted to assess the effectiveness of a tailored EBP-training program for Filipino physiotherapists, in improving knowledge, skills, attitudes and behaviour to EBP. Participation in this program aims to improve capacity to EBP and engage with referring doctors to determine the most effective treatments for their patients.

**Methods/Design**

A double blind randomised controlled trial, assessing the effectiveness of the EBP training intervention, compared with a waitlist control, will be conducted. An adequately powered sample of 54 physiotherapists from the Philippines will be recruited and randomly allocated to EBP intervention or waitlist control.

Intervention: The EBP program for Filipino physiotherapists is a one-day program on EBP principles and techniques, delivered using effective adult education strategies. It consists of lectures and practical workshops. A novel component in this program is the specially-developed recommendation form, which participants can use after completing their training, to assist them to negotiate with referring doctors regarding evidence-based treatment choices for their patients.

Pre and post measures of EBP knowledge, skills and attitudes will be assessed in both groups using the Adapted Fresno Test and the Questions to EBP attitudes. Behaviour to EBP will be measured using activity diaries for a period of three months.

**Discussion**

Should the EBP-training program be found to be effective in improving EBP-uptake in Filipino physiotherapists, it will form the basis of a much needed national intervention which is contextually appropriate to Filipino physiotherapists. It will therefore form the genesis for a model for building EBP capacity of other health professionals in the Philippines as well as physiotherapists in developing countries.

**Trial Registration**

Current Controlled Trials: ISRCTN74485061

## Background

Evidence-based practice (EBP) has been defined as the *"conscientious, explicit and judicious use of current best evidence in making decisions about the care of individual patients" *[1]. The use of research evidence in clinical practice has been an area of interest in the health profession around the world [2,3]. The aims of using evidence to guide practice are to build a defensible body of clinical professional knowledge, and use the best available evidence to support effective practice in patient care [4]. Strategies have been continuously proposed and tested to enhance the application of research evidence in practice. However, across all health professions, the uptake of the evidence into clinical practice has been a common challenge, because to do so requires change and sustainable thinking, and behaviours [2]. Barriers such as lack of knowledge, skills, time, and access to the literature have been consistently reported [5-8], and strategies are continually being refined to address the range of barriers that confront clinicians wanting to implement research evidence into practice.

In developing countries, there are additional barriers in applying evidence into guide clinical practice. The diversity and culture of these countries have had significant impact on the type of health care provided, and the way in which it is delivered to patients. Generalising evidence developed in other countries may not always be appropriate, and summary evidence from systematic literature reviews is often not relevant and applicable to developing countries. For instance, a recent Cochrane review reported that there is no evidence that using tap water for wound cleansing increases infection [9]. There are no trials included in the review to represent the use of tap water in developing countries and thus, the evidence cannot be recommended in settings where a constant supply of potable tap drinking water is unavailable. Consequently, context-specific clinical trials that can represent scenarios in developing countries can also be difficult to conduct, due to limited knowledge, resources, funding, skills etc. Trials conducted in developing countries have often been found to be of poor quality because of lack of research training of the authors. Thus these trials may well be excluded in international systematic literature reviews, as they may not meet the criteria of high methodological quality [10]. As a result, out-of-date and even harmful practices are still being implemented in developing countries, despite evidence to the contrary from international research [11].

There is no simple answer to this scenario. However, a key solution to overcome these barriers has been considered to be effective education, training and peer support [10-13]. Researchers and clinicians in developing countries need more support to learn the concepts and acquire the skills related to evidence based practice, so that research and clinical practices improve. Thus there is an urgent need to build capacity for EBP knowledge and skills amongst health professionals in developing countries. The South East Asia Optimising Reproductive and Child Health in Developing Countries known as the SEA-ORCHID project, is one of the few research-based projects which builds on this need to train health professionals (doctors, nurses and midwives) in evidence-based practice [13,14]. This group tested the effectiveness of a multifaceted intervention designed to increase the capacity for EBP and research synthesis, of the health professionals involved in reproductive medicine. The authors reported that although there were difficulties encountered during the project, such as access to academic expertise when the health professionals returned to their countries and availability of resources (i.e. computers with internet connection), the project resulted in a greater understanding of EBP and development of skills related to EBP.

The impact of using evidence to guide clinical practice is also being explored in the field of allied health. Allied health has been defined by the United States Association of Schools of Allied Health Professionals (ASAHP) as "*professionals [being] involved with the delivery of health care related services pertaining to the identification, evaluation and prevention of diseases and disorders; dietary and nutrition services; rehabilitation and health systems management, among other*..." [15]. The National Health Service (NHS) Scotland classified allied health as "scientific, technical and therapy workers" [16]. More recently, a broader and three-dimensional classification of Australian allied health has been presented by Turnbull et al [17]. This classification is built on allied health groupings of therapies, diagnostic and technical, scientific and complementary services. Physiotherapy is an allied health profession classified in the therapy group, along with occupational therapy, psychology, podiatry etc. These professions provide treatment to manage a range of conditions, particularly musculoskeletal, neurological and respiratory. As developing countries rarely have either enough money to fund adequate health services, or enough health professionals to provide adequate treatment, treatment interventions should be underpinned by the concepts of EBP to ensure efficient, and effective services.

The application of the concepts of EBP in physiotherapy can be challenging. There are issues which concern the generalisability of the evidence from the literature to the population of interest and the nature of practice in some countries where physiotherapists are not first-contact practitioners. Based from unpublished works, medical doctors in the Philippines who specialize in rehabilitation medicine make decisions regarding the type of physiotherapy care that should be provided, and physiotherapists by tradition, follow these instructions [18]. The strong influence of this tradition on the nature of physiotherapy practice frequently makes it difficult for physiotherapists in the Philippines to modify their usual way of thinking and treating. Barriers have been consistently reported in the Western world that are specifically related to uptake of EBP by allied health practitioners, including lack of knowledge, skills, time, and access to the literature [5-8]. Anecdotally, these barriers are broadly at play in the Philippines. Moreover, in addition to cultural practices, updating current knowledge and practice for Filipino medical doctors and therapists is difficult due to lack of access to research information [12] that is directly applicable and practical to patients within the context of the local health care system.

Specific to the needs of a group of allied health professionals, namely physiotherapists in the Philippines, we designed and pilot-tested an EBP training program to enhance their knowledge, skills, attitudes and behaviour to EBP. This tailored, multifaceted EBP program builds on the concept of effective education, training and peer support as key solution to overcome barriers to EBP in developing countries. It is further underpinned by evidence from the literature on contents and strategies of effective EBP programs [19-22]. We conducted preliminary exploratory studies which established the nature and scope of physiotherapy practice in the Philippines, the needs of Filipino physiotherapists and their perspectives on teaching EBP to the Filipino physiotherapy population [18].

The planned intervention (the EBP program for Filipino physiotherapists) is a one-day evidence-based program using effective adult education strategies. It consists of short lectures, practical workshops and practice sessions. A detailed description of the program is attached in this protocol. The training program has been pilot-tested and assessed for feasibility and acceptability. The training program resulted in a large effect size (0.83) in terms of improving knowledge and skills to EBP. The participants reported in evaluation summaries and feedback, that the content of, and strategies used in, the training were effective and practical. This training was viewed by participants as a medium to update physiotherapy practice and bridge the gap between theory and actual practice.

## Methods

### Study Aims

There are two aims in this research project:

1. To assess the effectiveness of the EBP training program in improving knowledge, skills, attitudes and behaviour of Filipino physiotherapists; and

2. To provide a tested model of continuing education and uptake of evidence which is contextually relevant to Filipino physiotherapists, and which could be adapted readily for physiotherapists from other developing countries, and allied health professionals who are not first contact practitioners in any health care system.

### Study design and setting

#### Ethics

Ethical approval has been obtained from the University of South Australia's Human Research Ethics Committee (protocol no. 0000021872). The primary author is a PhD student at this university.

#### Design

The EBP training program will be tested using a randomised controlled design, using a waitlist control group.

#### Outcomes

Significantly improved knowledge, skills, attitudes and behaviour to EBP as measured by psychometrically sound instruments. A moderate effect size of >0.6 of the education program is anticipated, based from several trials synthesized in a systematic review [23].

#### Timing

The study will commence in January 2011 and finish in October 2011. A detailed time table is attached with this protocol.

#### Sample Size determination

To detect a "moderate" effect size (0.6) at 80% power and 5% level of significance (2 tailed distribution), the required sample size for each group (intervention and control) is 27 physiotherapists (calculated using standard sample size calculator software in MedCalc). We will test for a conservative (moderate) effect in our RCT, as we are uncertain whether the large effect size from the pilot study was an artefact of the small sample, or an example of a Hawthorne effect.

#### Participant recruitment

Physiotherapists from hospitals and clinics in the Philippines will be recruited. Participants will be selected using the following criteria:

1. Licensed to practice physiotherapy and trained in the Philippines

2. Have not ever participated in any formal EBP training

#### Identification of reference population of participants

A register will be collated from a) a database of physiotherapists collated by the primary researcher from a preliminary study which established the profile of physiotherapists [18] and b) the registry of the Philippine Physiotherapy Association (PPTA), of which the primary researcher is a member and from whom approval has been sought and granted.

Physiotherapists who will express their interest in participating in the study will complete an information sheet which includes age, gender, years in practice, and highest educational attainment. There is also a screening component of the questionnaire which will seek information on any EBP training program. This section is particularly important as participants should not have been exposed to a formal EBP course or training to minimize the potential influence of such to the outcomes. To be specific, they should not have attended lectures and workshops on clinical question formulation, database searching, critical appraisal and applying findings on patient cases. Once screened for eligibility, participants will be asked to attend a meeting to orient them to the procedures of the study and to obtain written informed consents.

#### Randomisation and blinding

An independent researcher will randomly allocate participants into either the EBP training group or a waitlist control group, using a computer generated random numbers. Allocation into treatment and control groups will be undertaken by an independent research assistant who is unaware of the study objectives and the participants. The schedule of training will depend on the group allocation, such that those in the intervention group will have their training earlier than the waitlisted control group. Participants and assessors of the study outcomes will be blinded to the allocation at all times.

#### Procedure

**Figure 1:**
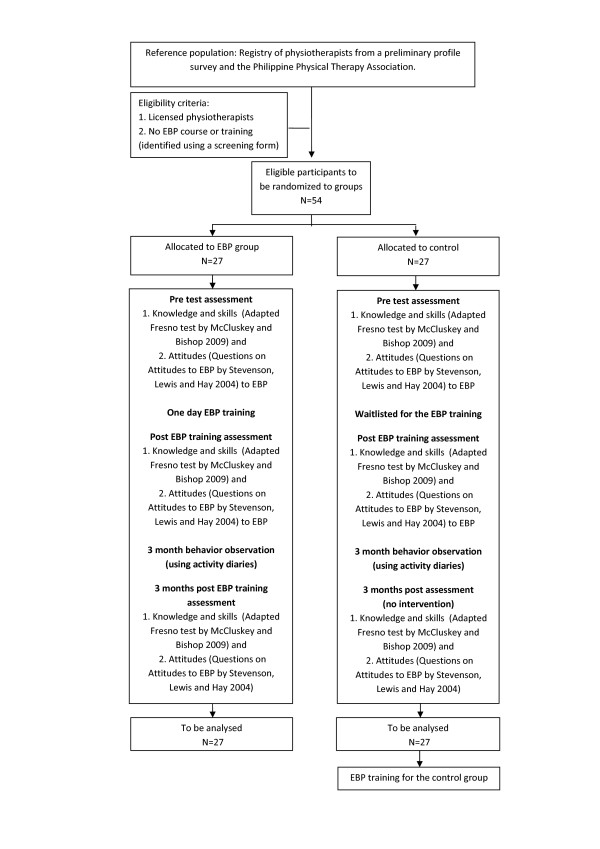
**Flow of procedures**.

All participants will be asked to answer the Adapted Fresno Test or the AFT [24] for baseline or pre- assessment of their knowledge and skills to EBP, and the Questions on attitudes to EBP [25], for baseline or pre- assessment of their attitudes to EBP (Figure 1). These tools will be sent through the participants' email, the day before the EBP intervention group's scheduled training.

**Figure 2:**
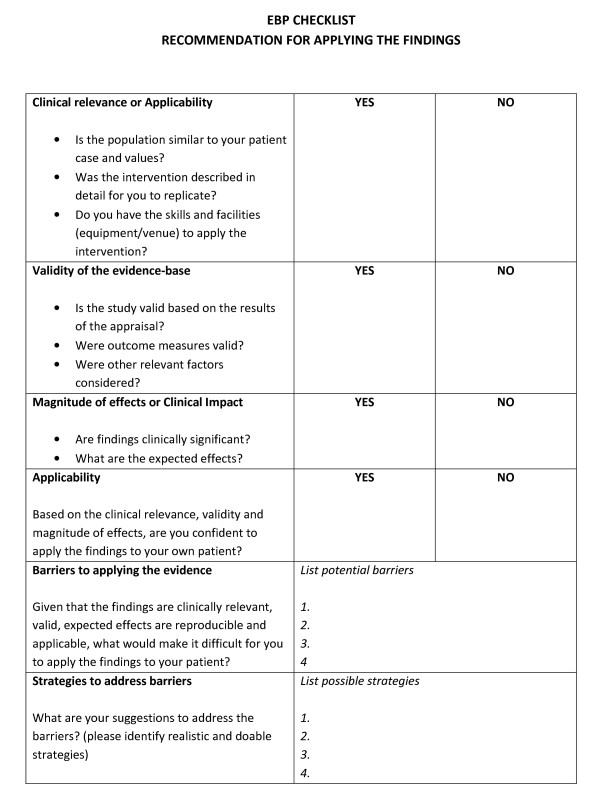
**EBP Checklist**.

The EBP intervention group will attend a one day training (Additional File 1) in a training centre at the University of Santo Tomas, Philippines. The objectives of the training will be provided to the participants before each of the six lectures and the practical sessions. Small group discussions will occur between the lectures, to enhance learning opportunities for participants. A checklist to assist participants to apply the evidence developed for this study will be provided as a take-home recommendation form, to guide the application of evidence in actual patient cases (Figure 2). After the EBP training, a link to an online support will be introduced to the participants. This has been developed to assist the participants in their EBP-related activities post training, as follow up support and multifaceted interventions have more potential to influence clinicians' behaviour to using evidence in practice [2]. This online support is further described in the intervention section of this document. Activity diaries will also be provided to participants to log their EBP-related activities when faced with a new or unique case, and provide their comments to their activities for a period of three months for short term EBP behaviour assessment.

At the end of the training, participants will be asked to provide a second set of answers to the Adapted Fresno test and the Questions on attitudes to EBP for post EBP training intervention assessment.

The waitlist control group will undergo the pre and post- knowledge, skills and attitude test at the same period as the intervention group. There will be no training for the control group during this time. However, for the next three months, they will also be provided with activity diaries to log their activities as basis for their treatment when faced with a new or unique case, whether these activities are EBP-related or not.

Three months post EBP training, a third set of answers to the Adapted Fresno test and the Questions on attitudes to EBP, will be obtained from both groups to measure if knowledge, skills and attitudes to EBP were sustained over the three month period in the EBP intervention group and to measure the stability of knowledge, skills and attitudes to EBP of the control group.

### The Intervention

The design of the EBP training program was based on preliminary exploratory studies on the nature and scope of physiotherapy practice in the Philippines, perspectives in teaching EBP to the local physiotherapists and the literature on effective training programs for allied health. From these studies, we identified culture specific strategies to teach Filipino physiotherapists about the concepts of EBP and how to apply them. The most effective training strategies appear to be short and simple lectures with plenty of time for practice sessions [22]. Educational strategies using combined lectures and workshops [26-29] which occur face-to-face and are conducted over at least a half-day are effective to improve knowledge and skills to EBP [22]. Multifaceted interventions and post training support have the potential to improve attitudes and behaviour to EBP, thus a post training online support has been designed for the participants. Principles of adult learning and educational strategies fitted to the type of population have also informed the design of the training program. Adult learners learn more by solving a problem [30] and so we designed the program in a manner where the participants will formulate their own clinical question and at the end of the training, should find an answer to the question based on the lectures and practice sessions provided.

The findings from our preliminary studies and the literature were layered in order to construct the **EBP for FilPTs **training program. The training program is composed of six lectures interspersed with four practical sessions to facilitate learning of knowledge and skills in EBP. The main contents of the training program are:

1. Introduction to EBP

2. Hierarchy of evidence and study designs

3. Drafting the clinical question using the PICO format

4. Designing the search

5. Critical appraisal of the evidence and

6. Answering the clinical question based from the evidence found.

The goal at the end of training is that the clinical question each participant formulated must be answered by the best available evidence. It is anticipated that the best evidence should be identified through a systematic literature search and is appraised as having good methodological quality. Small group discussions will occur during the training sessions to enhance participant learning. The **EBP Checklist **will assist participants to apply the evidence in practice. This will provide the 'tale-home' elements of the training as it can be used by participants to implement the evidence they have found in actual patient cases (Figure 2). This checklist is an innovative concept which physiotherapists who are not first contact practitioners, can use to recommend an evidence-based intervention to doctors for the management of a patient. The EBP checklist consists of items related to applicability or clinical relevance, validity of the evidence base, clinical impact or magnitude of effects. The EBP checklist is comparable to the Relevance, Education, Applicability, Discrimination Evaluation, Reaction (READER) checklist used by general physicians in evaluating the literature to make recommendations for treatment [31]. It is also based partly on the body of evidence matrix of the National Health and Medical Research Council (NHMRC) of Australia [32]. Additional items related to identifying potential barriers to applying the evidence with strategies to address the barriers were also included in this checklist. Barrier and strategy identification in the early stages help facilitate the process of evidence implementation [2].

This EBP checklist will be used by physiotherapists in making recommendations to referring doctors for the treatment intervention. For instance, if there is strong evidence that mobilization decreases pain among patients with chronic neck pain using the pain visual analogue scale (VAS), and the physical therapist has the skills to apply these mobilization techniques, this intervention can be recommended to the referring doctors using the EBP checklist. Mobilization may then form part of recommended treatment procedures, and its effectiveness assessed using a VAS. This process supports the application of principles of EBP, based on research evidence, the physiotherapists' skills and clinical expertise and the patient's goals, within the context of local practice. This process will enhance the decision making skills of the therapists, build their capacity as health professionals and assist them to work with other health professionals in an evidence-based manner.

An EBP online support designed as part of the education model will be made available to participants and can be accessed at this link: http://www.unisa.edu.au/cahe/Resources/EBPPhil/default.asp

The EBP online support allows participants the following:

1. Access the lectures and reference materials from the training (if participants need to review the slides, refresh their knowledge etc)

2. Seek assistance in searching for the evidence to answer their clinical question by filling up the PICO form

3. Seek assistance in validity assessment, through critical appraisal of the evidence found

4. Ask any queries and provide feedback

This follow-up online support complements the training provided to the physiotherapists and is a strategy to address previously identified barriers to evidence-uptake. Multiple strategies and tailored interventions to address barriers are more likely to facilitate changes in behaviour than single interventions [33,34].

### Outcome measures

#### EBP knowledge and skills

The Adapted Fresno Test will be used to measure EBP knowledge and skills. The test was adapted by McCluskey and Bishop [24] from the original Fresno test used in medicine by Ramos et al [35], and has been reported to have acceptable psychometric properties, in measuring change in knowledge and skills to EBP among novice rehabilitation professionals. The test consists of seven questions which address EBP competencies relevant to health practitioners. There are two versions of the test (different clinical scenarios for each test) and ICC scores for subtests were, Version 1: ICC, 0.80-0.96; Version 2: 0.68-0.94 and ICC for total scores were Version 1: ICC, 0.96; Version 2: ICC, 0.91. Internal consistency was acceptable (Cronbach's alpha = 0.74). For our study, version 1 was used for baseline assessment and version 2 for post training assessment. This choice was made to avoid any potential practice effect (defined as "*improvements in cognitive test performance due to repeated evaluation with the same test materials*"). Such practice effects can be a source of error [36].

Inter-tester reliability was assessed among the researchers participating in this study as part of the pilot study of the training program, and acceptable reliability was found (ICC 0.99) for both versions.

#### Questions to EBP attitudes

The Questions to EBP attitudes [25] will be used to assess the attitudes domain of EBP in this study. The attitudes to EBP scale consists of seven questions which are answered using a 3-point Likert scale: agree, neither agree nor disagree and disagree. The scale has been developed and validated for content and ease of completion [25]. In addition to this, participants will provide their comments on EBP and the EBP training program as part of this assessment.

#### EBP behaviour

EBP behaviour will be measured in two ways: (1) activity diaries to be provided to all participants, both intervention and waitlist groups, in paper and electronic copies, and (2) frequency of logging in, in the online support for the EBP intervention training participants http://www.unisa.edu.au/cahe/Resources/EBPPhil/default.asp, considered as an electronic EBP learning portfolio. As it is not possible to conduct actual participant observation in practice, activity diaries are commonly used to log and report participants' activities in EBP [37]. The electronic EBP learning portfolio on the other hand, which according to the literature, are both educational intervention and an evaluation strategy," *is the most promising approach to document actual performance of EBP steps *"[37].

The frequency of conducting non-EBP (asking a peer, reviewing a textbook etc) and EBP-related activities (drafting a clinical question in PICO form, searching the literature for the evidence, undertaking critical appraisal and applying the evidence found) will be logged in the activity diaries provided to the participants. The frequency of conducting these activities and the frequency of logging in the online support provided will be counted and interpreted as behaviour measures. Behaviour measures will be interpreted in two ways, evidence-seeking behaviour and practice-change behaviours ref. Activities which relate only to drafting a clinical question, searching and appraising the evidence, are classified as evidence-seeking behaviours. Applying the evidence to an actual patient case is classified as a measure of practice-change behaviour [38].

### Plan of analysis

Baseline statistical comparisons will be performed to ensure that all participants from both groups are homogenous and therefore comparable. All post-intervention analyses will be undertaken using an intention to treat approach. Within- and between-group analyses will be undertaken to measure pre and post intervention differences in both intervention and waitlist control group. Changes in outcomes will be compared for both groups using means and 95%Confidence Intervals. Time period for analyses will be pre-intervention, post intervention and three months post intervention.

## Discussion

We hypothesize that the EBP training will result in significant gains in knowledge, skills, attitudes and behaviour to EBP. More so, the EBP checklist designed for making treatment recommendations to doctors is envisioned to facilitate an EBP approach to providing best care by physiotherapists who are not yet first contact practitioners. In effect, this project will provide a tested model for building EBP capacity of physiotherapists, which can be utilized by other health professionals in developing countries, in establishing their credibility to deliver treatments that are evidence-based. Should our proposed study establish the effectiveness of an EBP training program tailored to meet the needs of physiotherapists in one developing country (the Philippines), it could be implemented more widely in the Philippines, and for physiotherapists in other developing countries. Such training may well have down-stream influences on reducing health care costs, and adverse events, because of a changing culture towards provision of evidence-based health care.

## Competing interests

The authors declare that they have no competing interests.

## Authors' contributions

All authors were responsible for drafting the research protocol and preparing the paper. JD, a PhD student, was responsible for designing the EBP training program with significant input from KG-S and SK. All authors read and approved the final manuscript.

## Pre-publication history

The pre-publication history for this paper can be accessed here:

http://www.biomedcentral.com/1472-6920/11/14/prepub

## Supplementary Material

Additional file 1 **Outline of the training program**. Click here for file
