# Supplementary material for: Effectiveness of the tailored EBP training program for Filipino physiotherapists: A randomised controlled trial
Source: BMC Med Educ. 2011 Apr 13;11:14. doi: 10.1186/1472-6920-11-14 (PMC3100239; doi:10.1186/1472-6920-11-14)
Supplement: Additional file 1 — Outline of the training program. [file 1472-6920-11-14-S1.DOC]

**Outline of the training program**

**EBP Training for Filipino Physiotherapists**

**Program Objectives**

The tailored Evidence Based Practice (EBP) program for Filipino physiotherapists aims to teach the concepts of EBP in a manner fitting the practice of physiotherapy in the Philippines. The EBP program also aims to develop evidence seeking and practice behaviour among the physiotherapists and therefore update their existing knowledge and enhance the use of the best evidence to guide practice in the long term process.

***Specific:***

1. To teach the concepts of EBP
2. To teach clinical question formulation using the PICO format (based on a scenario where clinician is faced with a new or unique case)
3. To teach participants how to search for the best evidence to answer the clinical question (including hierarchy of evidence and sources of the best available evidence)
4. To teach critical appraisal skills to assess the quality of the evidence found
5. To apply the knowledge and skills learned to draft an answer the question identified
6. To assess the process from question formulation to applying the skills learned

**Strategies**

Lecture and demonstration
Actual practice of skills
Problem solving
Post training support

**Assessment**

To objectively measures changes in EBP knowledge, skills and attitudes, the Adapted Fresno Test (McCluskey and Bishop 2009) and the Questions on EBP Attitudes (Stevenson, Lewis and Hay 2003) will be administered before and after the EBP program.

Activity diaries would be provided for participants to log activities (within 3 months post training) related to searching for answers to new or unique clinical cases.

| **Schedule** | **Topic** | **Strategy** | **Participant’s Output** |
| --- | --- | --- | --- |
| 08:00 – 08:30 | Orientation to the training |  |  |
| 08:30 – 09:00 | Introduction to EBP | Lecture 1 |  |
| 09:00 – 09:45 | Hierarchy of evidence and study designs | Lecture 2 |  |
| 09:45 – 10:00 | Break |  |  |
| 10:00 – 10:30 | PICO Formulation | Lecture 3  Practical session 1:  a. Drafting PICO from a case  b. Drafting own PICO | Own PICO |
| 10:30 – 11:15 | Designing the search | Lecture 4 with demonstration | Own search strategy |
| 11:15 – 12:00 | Conducting the search | Practical session 2 | Acquire evidence |
| 12:00 – 01:30 | Lunch |  |  |
| 01:30 – 03:00 | Appraising the evidence | Lecture 5  Practical session 3 | Appraised paper |
| 03:00 – 03:15 | Break |  |  |
| 03:15 – 04:30 | What does the evidence say and how applicable is it?  How can it be applied?  Use of the EBP checklist | Lecture 6  Practical session 4 | Answer to own PICO |
| 04:30 – 04:45 | Introduction to the online support | Demonstration |  |
| 04:45 – 05:00 | Post program knowledge, skills and attitudes assessment | Adapted Fresno test,  Questions on attitudes to EBP and answer evaluation questions |  |
